# A Kinematic Sensor and Algorithm to Detect Motor Fluctuations in Parkinson Disease: Validation Study Under Real Conditions of Use

**DOI:** 10.2196/rehab.8335

**Published:** 2018-04-25

**Authors:** Alejandro Rodríguez-Molinero, Carlos Pérez-López, Albert Samà, Eva de Mingo, Daniel Rodríguez-Martín, Jorge Hernández-Vara, Àngels Bayés, Alfons Moral, Ramiro Álvarez, David A Pérez-Martínez, Andreu Català

**Affiliations:** ^1^ Research Department Consorci Sanitari del Garraf Sant Pere de Ribes Spain; ^2^ Technical Research Centre for Dependency Care and Autonomous Living Universitat Politcnica de Catalunya Vilanova i la Geltru Spain; ^3^ Sense4Care Barcelona Spain; ^4^ Geriatrics Department Consorci Sanitari del Garraf Sant Pere de Ribes Spain; ^5^ Department of Neurology Hospital Universitari Vall D'Hebron Barcelona Spain; ^6^ Unidad de Parkinson y trastornos del movimiento Hospital Quirón-Teknon Barcelona Spain; ^7^ Department of Neurology Consorci Sanitari del Garraf Sant Pere de Ribes Spain; ^8^ Department of Neurology Hospital Universitari Germans Trias i Pujol Barcelona Spain; ^9^ Department of Neurology Hospital Universitario 12 de Octubre Madrid Spain

**Keywords:** Parkinson disease, movement disorders, movement, gait

## Abstract

**Background:**

A new algorithm has been developed, which combines information on gait bradykinesia and dyskinesia provided by a single kinematic sensor located on the waist of Parkinson disease (PD) patients to detect motor fluctuations (On- and Off-periods).

**Objective:**

The goal of this study was to analyze the accuracy of this algorithm under real conditions of use.

**Methods:**

This validation study of a motor-fluctuation detection algorithm was conducted on a sample of 23 patients with advanced PD. Patients were asked to wear the kinematic sensor for 1 to 3 days at home, while simultaneously keeping a diary of their On- and Off-periods. During this testing, researchers were not present, and patients continued to carry on their usual daily activities in their natural environment. The algorithm’s outputs were compared with the patients’ records, which were used as the gold standard.

**Results:**

The algorithm produced 37% more results than the patients’ records (671 vs 489). The positive predictive value of the algorithm to detect Off-periods, as compared with the patients’ records, was 92% (95% CI 87.33%-97.3%) and the negative predictive value was 94% (95% CI 90.71%-97.1%); the overall classification accuracy was 92.20%.

**Conclusions:**

The kinematic sensor and the algorithm for detection of motor-fluctuations validated in this study are an accurate and useful tool for monitoring PD patients with difficult-to-control motor fluctuations in the outpatient setting.

## Introduction

Parkinson disease (PD) is the second most frequent neurodegenerative disease after Alzheimer, with an age-standardized annual incidence rate of 160 per 100,000 subjects aged 65 years or older [1]. Patients suffering from this disease present a motor disorder characterized by muscle stiffness (rigidity) and slow movement (bradykinesia), sometimes accompanied by tremor and freezing of gait (patients feel their feet are stuck to the ground and cannot take a step). These symptoms are related to a deficiency in neurotransmitter dopamine in certain brain areas that are in charge of the motor control. Therefore, exogenous administration of L-Dopa or other dopaminergic agonists constitutes the first line of treatment in PD [2]. As the disease progresses, patients experience motor fluctuations between a so-called On-state, where symptoms are under control and the patient can move fluently, and a so-called Off-state, where motor symptoms reappear or worsen [3]. It is currently considered that Off-periods are related to the waning of dopaminergic medication effects and that they can be relieved by keeping stable plasmatic levels of medication. Thus, dose fractionation, prolonged-release preparations, or drug infusion pumps can be used with the aim of providing more physiological continuous dopaminergic stimulation. Furthermore, patients with advanced PD may present dyskinesia—involuntary and excessive movement of one or more body segments—which is related to excessive dopaminergic stimulation and, again, may be ameliorated by keeping plasmatic dopaminergic drugs at a stable level [4].

To make appropriate therapy adjustments to reduce motor fluctuations and dyskinesia, physicians need detailed information on the time course of these symptoms, which may appear several times a day. Due to the fluctuating and irregular nature of motor manifestations, such information is hard to collect in office. Thus, physicians may ask patients to keep written records of the times of the day when fluctuations occur. However, such records have severe limitations, in terms of the quality of the collected information, due to memory bias and low patient adherence [5]. Therefore, devices capable of automatically and continuously detecting and recording motor fluctuations would be very welcome in the clinical practice; they could help physicians optimize therapy schedules, thus, enhancing patients’ quality of life. Furthermore, they would be extremely useful tools in clinical trials for new therapies, where the basic evaluation parameter is the time in Off state, which is very difficult to measure reliably and uniformly by other means.

Inertial sensors, especially accelerometers, have been used to detect and quantify various motor symptoms of PD. Zwartjes et al studied the severity of bradykinesia, hypokinesia, and tremor in 6 PD patients using 4 inertial sensors (located on the wrist, thigh, foot, and sternum) and found a correlation between their measurements and the Unified Parkinson's Disease Rating Scale (UPDRS) [6]. Salarian et al measured tremor and bradykinesia in 10 patients, using an accelerometer on each forearm and also found a good correlation with the UPDRS [7]. During the course of the European project PERORM [8], a system with 5 sensors was developed, which classified the severity of bradykinesia, tremor, and dyskinesia with 87% accuracy. Keijsers et al detected dyskinesia in 6 patients, using 3 inertial sensors, with 96.6% accuracy [9]. Other authors have used inertial sensors to analyze the freezing of gait [10-12], although the accuracy of their detection algorithms was lower, often below 70%. A few authors have attempted to detect motor fluctuations (On and Off-periods) rather than individual symptoms [13-15]. However, to the best of our knowledge, at present there is no device available in the market or being tested in research studies capable of detecting motor phases (On and Off) frequently and accurately enough to help physicians adjust the dopaminergic medication regimen. Our research team developed an accelerometry-based sensor device and the corresponding algorithm, which can make frequent readings of the patient’s motor state, with the aim of providing a useful tool for therapeutic schedule adjustments [16]. The goal of this study was to analyze the accuracy of the kinematic sensor and the algorithm under real conditions of use, in a group of PD patients with motor fluctuations.

## Methods

### Participants

This prospective validation study was conducted on a sample of 23 patients with moderate to severe PD and motor fluctuations. Patients unable to recognize different motor states (On and Off), presenting gait disorders other than those of PD, or unable to walk without the help of a third person were excluded.

The study was conducted entirely in the province of Barcelona, Catalonia (Spain), between 2013 and 2016. Participants were selected by convenience sampling among those attending the neurology clinics of any of the 4 participating hospitals (Consorci Sanitari del Garraf, Centro Médico Teknon, Hospital de Vall d'Hebron, Hospital Germans Trias i Pujol), or among the members of the Catalonian Association for Parkinson (Asociació Catalana Per al Parkinson) and its subsidiaries. The sample size was chosen on the basis of previous experience in similar validation studies [8,13,17].

### Data Collection

**Figure 1:**
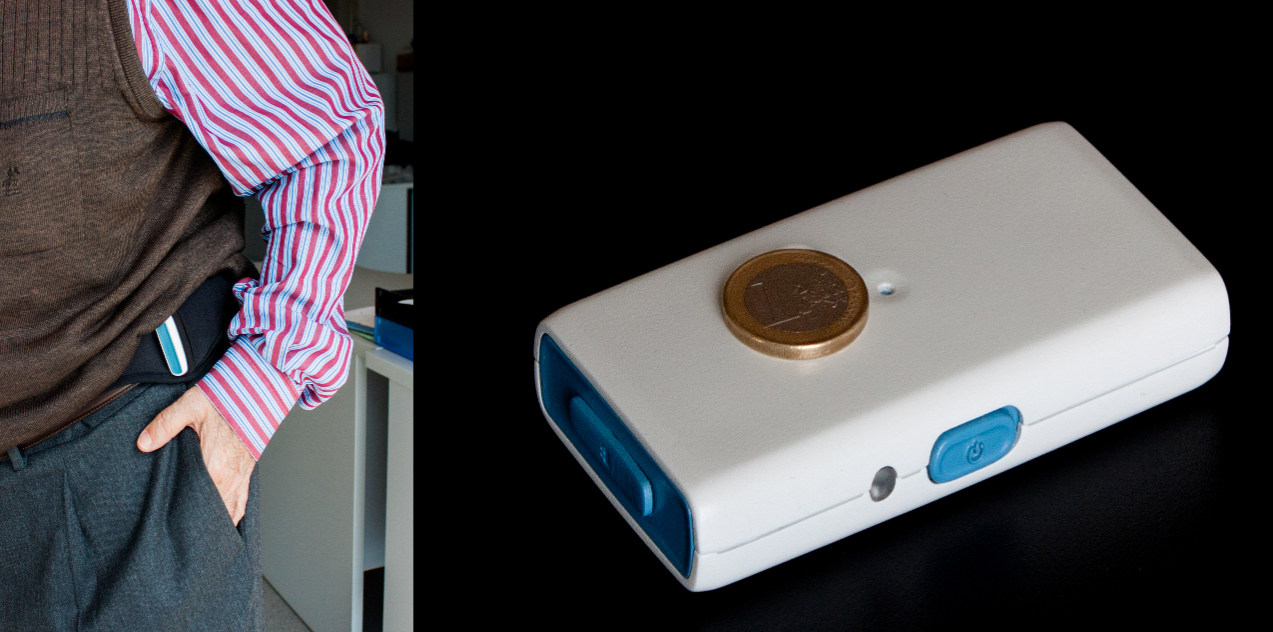
Inertial sensor.

On the first day of the study, all patients were administered the motor section of the UPDRS [18]. Sociodemographic data (sex, age, and marital status), years of evolution of the PD, and the drug regimen were also recorded. Participants were asked to wear the kinematic sensor attached to the waist (left lateral side) for a variable number of daytime hours, within a period of 1 to 3 consecutive days, according to their individual preference. The location on the waist was comfortable for patients and suitable to provide precise information about the body movements [19-21] (Figure 1).

During the study, patients were living in their usual environment, carrying on their usual daily activities, and were not suggested or prevented from doing any specific task. Patients were also asked to simultaneously keep a specially designed diary, where they had to record their motor state (On or Off) every 30 min. All patients were previously instructed regarding the use of the diary and the recognition of their motor fluctuations. Patients were blind to the records provided by the sensor they were wearing. Researchers were not present during the time patients were wearing the sensor in order to prevent interferences in their natural activities. However, a researcher was in charge of calling them by telephone every 2 to 3 hours to reinforce the use of the diary and record the motor state reported by the patient at the moment of the telephone call. The sensor and its battery charger were handed over on the first day and collected on the last day of the study. The patients or their accompanying person were in charge of recharging the device during the hours it was not being used. Local Ethics Committees approved the research protocol at each participating institution. All participants signed an informed consent form before their inclusion in the study.

### Algorithm Overview

The sensor readings were based on measurements from the accelerometer—sampled with a 40 Hz frequency—and provided output in nonoverlapping 10-min periods. The output of every 10-min period consisted in: presence or absence of gait bradykinesia plus presence or absence of dyskinesia.

Since patients in the Off-state do not present choreoic-type dyskinesia; detection of dyskinesia was considered an indicator of the On-state. Failure to detect dyskinesia left the classification of the motor state to the presence or absence of bradykinesia: Off-state for clearly bradykinetic gait, On-state for normal gait, and intermediate-state for abnormal gait that however did not reach the threshold to be considered bradykinesia. Failure to detect any movement (neither dyskinesia nor gait) led us to consider the motor state unknown. The algorithmic process to detect bradykinesia and dyskinesia has been described in detail elsewhere [16]. However, a short description is offered below for the sake of self-completeness.

Briefly, a first algorithm was designed to analyze patients’ bradykinetic gait in the following 5 phases:

1. Walk detection was based on 3.2-sec signal segments, which were characterized by their power spectra and analyzed with a support vector machine (SVM). The SVM had been previously trained with labeled signals from 20 different PD patients, who did not participate in the On and Off state monitoring for data collection in this study. Walk detection accuracy was higher than 90% [23].
2. Stride detection was carried out on those signal segments, on which the SVM detected that the patient was walking. It was based on biomechanical properties reflected in the acceleration signals; namely, every time the patient took a step (the so-called initial contact event, when the foot touches the ground) a local relative extremum was observed in the 3 acceleration signals, which was leveraged to identify the strides. The first two and the last two strides in a walking bout were disregarded in order to avoid analyzing gait initiation and finalization.
3. Characterization of every stride in terms of movement fluidity—a feature closely related to bradykinesia, which the authors found to be correlated with the On and Off-states in a previous study [24]. This feature consisted in the power spectra within the 0 to10 Hz band of the acceleration measurements comprising a stride. In this way, by providing the detected strides (once gait initiation and finalization strides had been disregarded), a scalar value representing movement fluidity was obtained. In our aforementioned earlier work, we found higher values for patients in the On-state and lower values for those in the Off-state.
4. Calculation of the average of all strides comprised in every nonoverlapping 10-min period.
5. Comparison of this average with the patient’s individual threshold. If the average fluidity in a 10-min period was higher than the threshold, gait bradykinesia was considered for that patient in that period; whereas if it was lower, bradykinesia was ruled out. Finally, if the average value was close to the threshold (within the range: threshold ± 1.7 m/s^2^) intermediate gait was considered.

**Figure 2:**
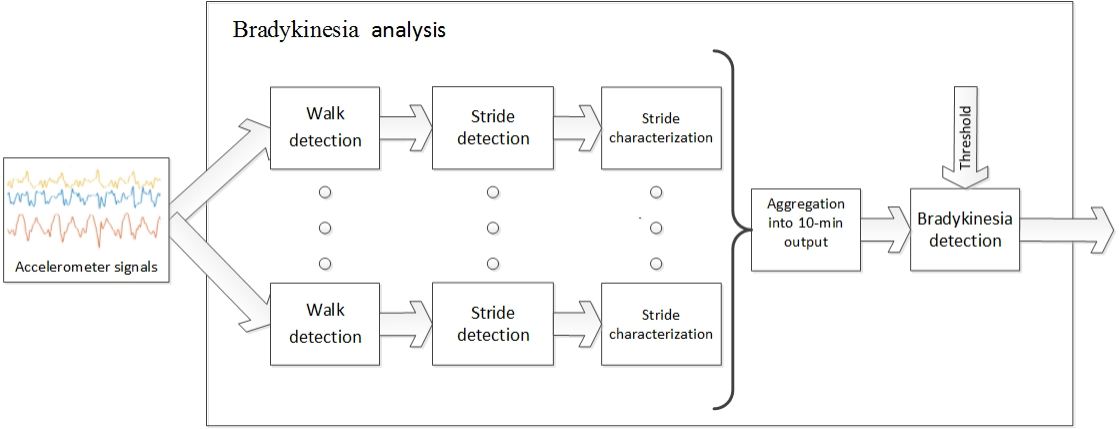
Bradykinesia analysis.

The algorithm’s final output was presence or absence of gait bradykinesia or indeterminacy, in case the subject had not walked within the analyzed 10-min period (Figure 2). The patient-specific threshold was established in an unsupervised manner based on the distribution of gait fluidity measurements recorded while monitoring each patient. In particular, the threshold was calculated by using the histogram of fluidity measurements. Thus, if 2 separated bell-shaped curves were observed, each one with at least 15% of data, the threshold was established at the mean value between them. However, if 2 bell-shaped curves were not obtained, or if they did not contain enough data, the bradykinesia threshold was established at the highest bradykinesia value below the mode with a frequency of at least 60% of the frequency of the mode.

A second algorithm was designed to analyze choreoic dyskinesia on the basis of the frequency content of the accelerometer measurements. This second algorithm was organized in the following phases:

1. Walk detection and postural transition detection, based on 3.2-sec acceleration signal segments, where the above mentioned SVM was used to determine whether the patient was walking, plus analysis of the power spectra between 0.1 and 0.6 Hz (by comparison with a previously determined threshold, which was the same for all the patients) to establish whether the patient was engaged in a postural transition (eg, stand-to-sit or sit-to-stand). In case walking or postural transition was detected in a signal segment, the following phase 2 was skipped, given that such actions were considered to possibly hide dyskinetic movements.
2. Dyskinesia detection. For every 3.2-sec segment in which the patient was not walking or in postural transition, the power spectra between 1 and 4 Hz were compared with a threshold (previously determined and the same for all patients) to assess whether the patient presented dyskinesia in that segment.
3. Aggregation per minute. If the ratio of segments, which were analyzed within a certain minute (ie, the ratio of segments without walking or postural transitions) was lower than 30%, the output of the algorithm for that particular minute was undetermined. If the ratio of segments with positive dyskinesia detection was higher than 40%, presence of dyskinesia was considered for that minute. Otherwise absence of dyskinesia was considered.
4. Finally, the output for periods of 10 nonoverlapping consecutive minutes was obtained. If 8 out of the 10 min in a certain period were undetermined, the output of that 10-min period was considered to be undetermined. Otherwise, presence or absence of dyskinesia was considered on the basis of the most frequent per-minute output in that period.

Patients’ motor state was estimated in 10-min periods, according to the output of the above described algorithms. The motor state was classified as On-state when the patient did not show gait impairment or showed dyskinesia, Off-state when the patient showed bradykinetic gait and did not show dyskinesia, intermediate-state when the patient showed intermediate gait and did not show dyskinesia, and any other situation was classified as unknown motor state. In patients, who did not show dyskinesia, the motor state was established according to bradykinetic gait. Finally, the 10-min periods were analyzed in groups of three consecutive ones. In case the outputs of the first and the third periods were equivalent and that of the second period was unknown, the output of the second period was considered to be the same as the first and the third ones.

### Training and Testing of the Machine Learning Algorithms

The training of the bradykinetic gait-detection algorithm, which corresponded to training the SVM for gait detection, was carried out with data from 20 PD patients from a previous research study [22]. The bradykinesia feature was identified in that study and did not require any training procedure as it merely characterized the strides. Finally, the threshold to be compared with the averaged bradykinetic features was calculated for each patient, according to the procedure described above (using the histogram of fluidity measurements), which did not require any machine learning algorithm and was applied in an unsupervised manner (ie, diaries were not used).

The dyskinesia-detection algorithm only required one supervised learning model, which was the SVM used in gait detection. Dyskinesia and postural transitions were detected by comparing the signal’s power spectra with specific thresholds, established in 2 previous research studies [22,24]. Finally, the thresholds for the analysis of the 1-min and 10-min periods had also been established in previous research studies and were used as constant values to analyze the signals from all patients.

In summary, the only part of the algorithm that was adapted to each patient was the bradykinetic gait threshold, which was unsupervised. The remaining parts of the algorithm were constant and had been established in previous research studies.

### Statistical Analysis

To evaluate the accuracy for classification of the algorithm readings, they were compared with the records on the patients’ diaries. Time slots with no information on a patient’s diary were excluded from the analysis. Accuracy was calculated by using the following formula:

Accuracy=(TP+TN)/(TP+TN+FP+FN)

where TP are true positives, TN true negatives, FP false positives, and FN false negatives.

Positive predictive value (PPV) and negative predictive value (NPV) were calculated by using the following formulas:

PPV=TP/(TP+FP)

NPV=TN/(TN+FN)

where TP are true positives, TN true negatives, FP false positives, and FN false negatives.

The sensitivity (number of Off-episodes detected out of the total Off-episodes occurred) and the specificity (number of On-episodes detected out of the total On-episodes occurred) were not calculated, as the actual number of Off and On-episodes cannot be found out in an unsupervised experiment (patients do not rigorously record all the motor-phases in their diaries, which is the reason why better outpatient monitoring standards are sought).

The raw accuracy measurements for the total sensor’s readings were calculated by comparison with the patients’ records, whenever they were available (for this calculation, patients with longer monitoring times contributed more to the result). Additionally, average accuracy measurements were calculated by calculating the accuracy for every individual patient and averaging the results (for this calculation, the data from every patient weighted the same) Statistical analysis was conducted with the SPSS V.21.0 software package (IBM Corp, Armonk, NY).

## Results

### Participant Data

Out of 32 initially contacted patients, 6 were excluded because they did not experience motor fluctuations or were unable to recognize their motor state, and one was excluded because of inability to walk. The data from 2 patients were not valid because of errors during the data collection process: sensor malfunction (data were deleted or recorded incorrectly) or misuse (not switching the sensor off properly or not noticing error messages). A total of 23 patients with complete datasets were eventually included. Table 1 shows the sociodemographic and health-related data from the 23 participants.

**Table 1:** Characteristics of the participants (N=23).

Characteristic		Statistics
Age in years, mean (SD)		63.8 (9)
Male, n (%)		16 (70)
Female, n (%)		7 (30)
Married, n (%)		16 (70)
Single or widower, n (%)		7 (30)
Years of disease, mean (SD)		9.8 (5)
Total L-Dopa dose (mg/day), mean (SD)		723 (486)
UPDRS^a^(motor section), median (IQR^b^)		21 (16)
**Percentage of daily time in Off-state**		
	1% to 25%, n (%)	16 (70)
	26% to 50%, n (%)	7 (30)

^a^UPDRS: Unified Parkinson's Disease Rating Scale.

^b^IQR: interquartile range.

**Table 2:** Sensor and algorithm’s validation results.

Patient	Positive predictive value (%)	Negative predictive value (%)	Accuracy (%)	Total sensor detections	Sensor output with gold standard available	Total diary annotations	Monitoring time (hours)	Number of monitoring days
1	80	83	82	19	11	10	11.2	1
2	N/A^a^	100	100	4	1	5	4.2	1
3	100	100	100	29	16	16	8.6	1
4	100	100	100	12	7	6	3.1	1
5	100	100	100	7	4	14	11.0	1
6	100	100	100	8	3	6	18.6	1
7	N/A	100	100	34	27	19	10.9	1
8	N/A	100	100	10	2	6	9.0	1
9	92	100	95	38	19	22	19.0	2
10	N/A	92	92	102	74	44	40.1	3
11	100	83	88	53	16	33	27.0	2
12	80	100	92	19	13	9	18.1	2
13	94	73	84	48	31	30	41.1	3
14	90	94	93	93	60	52	40.4	3
15	67	100	83	23	12	25	27.9	2
16	100	84	85	34	27	24	41.0	3
17	100	91	93	37	27	25	35.2	3
18	100	95	96	42	24	48	36.3	3
19	67	100	71	11	7	10	13.4	2
20	N/A	92	92	19	12	21	39.3	3
21	100	71	75	17	8	48	35.2	3
22	100	100	100	9	7	13	24.2	2
23	N/A	100	100	3	2	3	10.0	1
Total	92	94	92	671	410	489	524.6	45

^a^N/A: not applicable.

### Evaluation Outcomes

The mean monitoring time was 23 hours per patient. During that time, a sensor produced 671 conclusive results (On or Off classifications or detections) for all patients, which corresponded to 22.8 results per patient and 1.3 On or Off classifications per hour and patient. From all the sensor detections, only 410 had a corresponding record in the patients’ diaries with which they could be compared. The sensor’s raw PPV, calculated by comparing the total of sensor readings with the total of patients’ records, was 89.3% (95% CI 85.8%-92.1%), the row NPV was 92% (95% CI 88.9%-94.4%). Table 2 shows the PPV and NPV for each individual patient. The mean of PPV and NPV for all the patients were 92% (95% CI 87.33%-97.3%) and 94% (95% CI 90.71%-97.1%), respectively. The average classification accuracy was 92.20%. Patients’ individual accuracies are also shown in Table 2.

**Figure 3:**
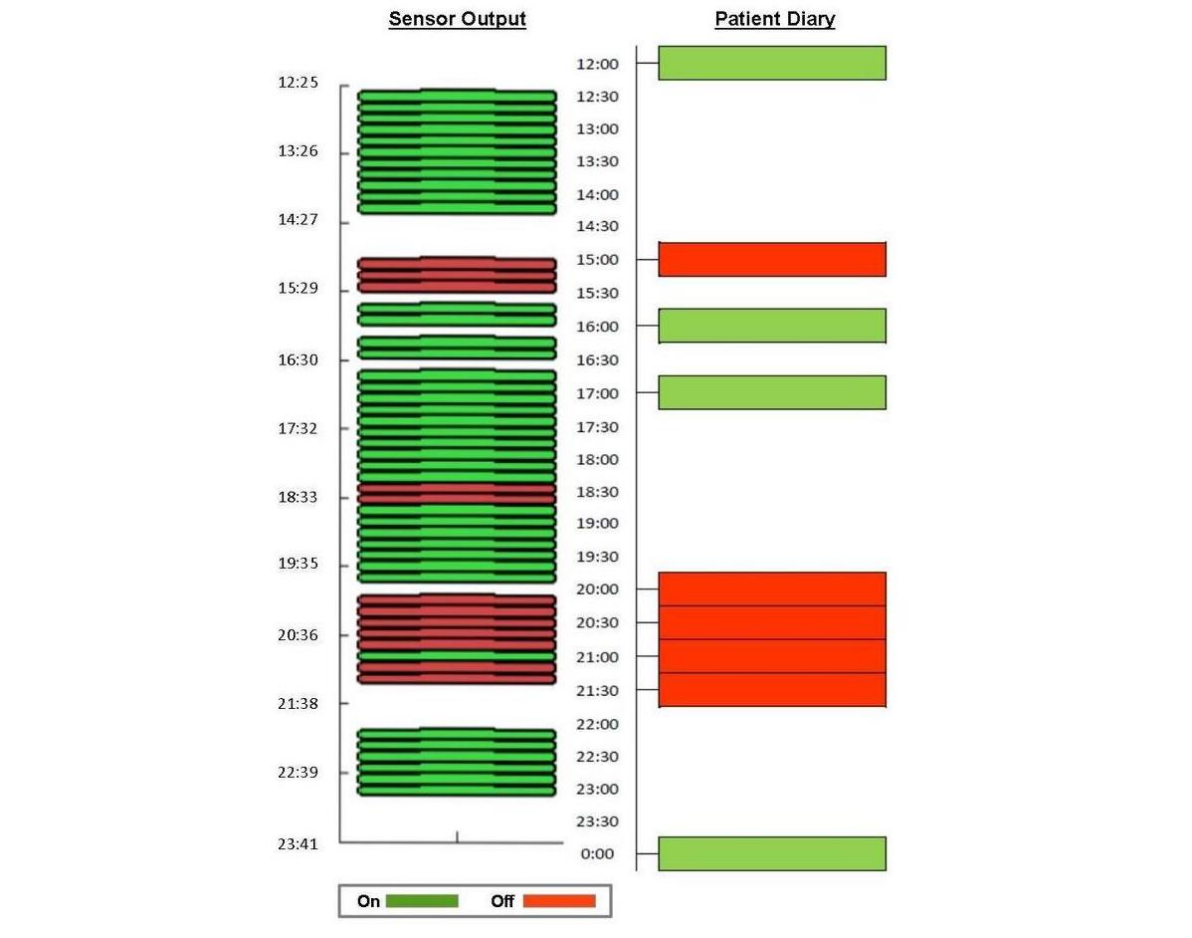
Comparison between the outcomes of the algorithm and the data recorded in a patient's diary.

Figure 3 shows an example of comparison between the outcomes of the algorithm and the data recorded in a patient’s diary.

## Discussion

### Principal Findings

Our results evidenced that the tested algorithm accurately detected motor fluctuations in patients with advanced PD. Additionally, as the experimental work was neither carried out in a controlled environment nor observed by researchers, the results might be extrapolated to the clinical practice. Experiments were conducted under real conditions of use and the data provided by the sensor were compared with the most extensively used standard in the clinical practice that is, the patients’ diaries. Given the limitations of this standard, we used it only in patients who were able to recognize their motor state and additionally provided telephonic reminders to reinforce its use. Note that previous calibration or adaptation of the algorithm to individual users was not necessary; instead, a self-calibration method was used, which avoided the need of conducting previous tests with the patients.

### Comparison With Earlier Evidence

As far as we know, only 2 research studies have been attempted to detect the On and Off-motor states under real conditions using inertial sensors. In one of these studies, the commercially available device Kinetigraph, a bracelet with an accelerometer, was found to detect bradykinesia and dyskinesia [17] and to classify fluctuating patients versus controls [25] with an acceptable accuracy. However, the device and its algorithms offered global results (over a certain period of time) and could not make hourly determinations of the motor state with good accuracy (correlation was .4 in a comparison with patients’ diaries) [13]. Therefore, though it might be useful to evaluate whether a patient’s time in Off-state is reduced (or not) by the medication in the medium term, it would not be useful to determine the times of the day when the patients are in Off-state with accuracy enough as to fine-tune therapeutic regimens. In the second study, Hoff et al used multichannel accelerometry, previously validated for the detection of hypokinesia, bradykinesia, and tremor. However, their measurements showed limited sensitivity (0.60-0.71) and specificity (0.66-0.76) for motor fluctuations in individual PD patients (the authors did not provide the percentage of classification accuracy) [14]. Keijsers et al used 6 sensors located on different parts of the body to detect motor fluctuations, although their experiment was conducted in laboratory instead of real conditions [15]. These authors analyzed the whole inertial signal produced over the 3 hours of the experiment (not only those moments when the algorithm produces an output as in our research); in such circumstances, sensitivity and specificity correspond to PPV and NPV, respectively. They found acceptable sensitivity and specificity for detection of the motor state through measurements of bradykinesia on the wrist (sensitivity 0.71-0.74; specificity 0.78) and leg (sensitivity 0.78; specificity 0.82). They did not use sensors on the waist but used one on the chest, which showed high accuracy (sensitivity 0.96; specificity 0.95) by measuring a thoracic tremor which was found to be greater in the Off-state than in the On-state, even in patients with nontremors PD. This finding is, however, difficult to interpret; and, as far as we know, it has not been reproduced in subsequent studies (including an attempt made by our team; not published).

Although, up to our knowledge, no further studies aimed at detecting the motor state have been published, other authors have tried to detect bradykinesia, which is related to the Off-state [6,26,27]. Most of such studies have been conducted in a laboratory or a clinic (controlled environment) in conditions of restricted activity or asking the patients to perform certain maneuvers, so that their results can hardly be directly extrapolated to the clinical practice. Salarian et al [7] allowed their subjects free activity for 3 hours (although inside a clinic instead of their own environment) and found a good correlation (Spearman rank correlation, ρ=.7) between the data from 2 Physilog sensors placed on the forearms and the bradykinesia item of the UPDRS. Tzallas et al [8] tested 5 sensors composed of accelerometers and gyroscopes and located on limbs and trunk in a real environment. Their algorithms showed a moderate accuracy (74.5% classification accuracy) for detection of bradykinesia as compared with the records made by the patients or their relatives.

### Limitations

Our algorithm requires the occurrence of movement (gait or choreoic dyskinesia) to be able to determine the motor phase. Thus, it does not continuously provide data; and there are time slots in which the motor state remains unknown. Therefore, undetected Off-periods are probable to occur, which would reduce the system’s sensitivity to detect Off-periods. In this experiment, we were unable to measure sensitivity (the number of detected Off-periods out of the total actually occurring Off-periods) because the chosen gold standard does not record all the Off-periods either (the patient may forget or fail to record some of them). However, we found high predictive value and accuracy, which means that whenever the sensor makes a determination, it is often correct. The time during which detections are not possible is an obvious limitation of this system. However, to see it in perspective, the sensor provides more information than the patient’s diary (which is the best method known to date). For example, the sensor in this study collected 37% more valid data (On-Off detections) than the patients’ diaries. Furthermore, patients may fail to complete the diary time records for several consecutive days because it is an arduous task and they often give up [5]. Note that, in our study, 6 patients voluntarily stopped data recording before the third day, due to the inconvenience of filling the diary (results not shown).

The results of validation studies of new monitoring systems for PD should be interpreted with caution, due to the limitations of the reference standards currently used [28]. Methods based on new technologies may be better than traditional methods such as a patient’s diary. Thus, outcome differences between both approaches may be more probably due to limitations of the standard than to poor validity of new technology methods. We postulate that, in this case, our aim should not be creating technologies as effective as traditional standards but overcoming these standards. Therefore, although concurrent validation studies are necessary (validation by comparison with a standard), prospective validation studies are needed, where the utility of new technologies to achieve better clinical control is demonstrated or ruled out.

In this study, we used patients’ diaries as the reference standard because no better alternative is currently available for long-term monitoring patients in their natural environment. However, it should be taken into account that patients recruited for this study were able to recognize their motor state well and received telephone reminders to complete the diary. In the clinical practice, many patients cannot actually recognize their motor state or fail to record it in the diary, all of which reduces the accuracy of the diary method and supports the development of automatic detection methods.

As previously reported [22], our system was able to detect dyskinesia and consequently, to distinguish between On-state with dyskinesia (which may reflect excessive dopaminergic stimulation) and On-state without dyskinesia (which indicates optimal stimulation). In this study, however, such a distinction was not validated because, given the experimental design (prolonged monitoring without direct observation), preparing a good standard to verify the presence of dyskinesia was not possible; dyskinesia involves involuntary movements of which patients often remain unaware.

### Conclusions

In conclusion, the kinematic sensor and the algorithm validated in this study constitute an accurate and useful tool for monitoring and recording motor fluctuations in patients with moderate-advanced PD in the outpatient setting.

## Abbreviations

FN: false negatives
FP: false positives
NPV: negative predictive value
PD: Parkinson disease
PPV: positive predictive value
SVM: support vector machine
TN: true negatives
TP: true positives
UPDRS: Unified Parkinson's Disease Rating Scale

## References

[1] Hirtz D, Thurman D, Gwinn-Hardy K, Mohamed M, Chaudhuri A, Zalutsky R (2007). How common are the “common” neurologic disorders?. Neurology.

[2] Kalia LV, Lang AE (2015). Parkinson's disease. Lancet.

[3] Tolosa E, Wenning G, Poewe W (2006). The diagnosis of Parkinson's disease. Lancet Neurol.

[4] Fabbrini G, Brotchie JM, Grandas F, Nomoto M, Goetz CG (2007). Levodopa-induced dyskinesias. Mov Disord.

[5] Papapetropoulos SS (2012). Patient diaries as a clinical endpoint in Parkinson's disease clinical trials. CNS Neurosci Ther.

[6] Zwartjes DG, Heida T, van Vugt JP, Geelen JA, Veltink PH (2010). Ambulatory monitoring of activities and motor symptoms in Parkinson's disease. IEEE Trans Biomed Eng.

[7] Salarian A, Russmann H, Wider C, Burkhard PR, Vingerhoets FJ, Aminian K (2007). Quantification of tremor and bradykinesia in Parkinson's disease using a novel ambulatory monitoring system. IEEE Trans Biomed Eng.

[8] Tzallas AT, Tsipouras MG, Rigas G, Tsalikakis DG, Karvounis EC, Chondrogiorgi M, Psomadellis F, Cancela J, Pastorino M, Waldmeyer MT, Konitsiotis S, Fotiadis DI (2014). PERFORM: a system for monitoring, assessment and management of patients with Parkinson's disease. Sensors (Basel).

[9] Keijsers NL, Horstink MW, Gielen SC (2003). Automatic assessment of levodopa-induced dyskinesias in daily life by neural networks. Mov Disord.

[10] Moore ST, MacDougall HG, Ondo WG (2008). Ambulatory monitoring of freezing of gait in Parkinson's disease. J Neurosci Methods.

[11] Delval A, Snijders AH, Weerdesteyn V, Duysens JE, Defebvre L, Giladi N, Bloem BR (2010). Objective detection of subtle freezing of gait episodes in Parkinson's disease. Mov Disord.

[12] Moore ST, Yungher DA, Morris TR, Dilda V, MacDougall HG, Shine JM, Naismith SL, Lewis SJ (2013). Autonomous identification of freezing of gait in Parkinson's disease from lower-body segmental accelerometry. J NeuroEngineering Rehabil.

[13] Ossig C, Gandor F, Fauser M, Bosredon C, Churilov L, Reichmann H, Horne MK, Ebersbach G, Storch A (2016). Correlation of quantitative motor state assessment using a kinetograph and patient diaries in advanced PD: data from an observational study. PLoS One.

[14] Hoff JI, van der Meer V, van Hilten JJ (2004). Accuracy of objective ambulatory accelerometry in detecting motor complications in patients with Parkinson disease. Clin Neuropharmacol.

[15] Keijsers NL, Horstink MW, Gielen SC (2006). Ambulatory motor assessment in Parkinson's disease. Mov Disord.

[16] Pérez-López C, Samà A, Rodríguez-Martín D, Català A, Cabestany J, Moreno-Arostegui JM, de Mingo E, Rodríguez-Molinero A (2016). Assessing motor fluctuations in Parkinson's disease patients based on a single inertial sensor. Sensors (Basel).

[17] Griffiths RI, Kotschet K, Arfon S, Xu ZM, Johnson W, Drago J, Evans A, Kempster P, Raghav S, Horne MK (2012). Automated assessment of bradykinesia and dyskinesia in Parkinson's disease. J Parkinsons Dis.

[18] Fahn S, Elton R (1987). Recent Developments in Parkinson’s Disease, Vol. 2.

[19] Yang CC, Hsu YL (2010). A review of accelerometry-based wearable motion detectors for physical activity monitoring. Sensors (Basel).

[20] Gjoreski H, Lustrek M, Gams M (2011). Accelerometer Placement for Posture Recognition and Fall Detection.

[21] Mathie MJ, Basilakis J, Celler BG (2001). A system for monitoring posture and physical activity using accelerometers. http://ieeexplore.ieee.org/document/1019627/.

[22] Samà A, Pérez-Lopez C, Romagosa J, Rodríguez-Martín D, Català A, Cabestany J, Pérez-Martínez DA, Rodríguez-Molinero A (2012). Dyskinesia and motor state detection in Parkinson's disease patients with a single movement sensor.

[23] Samà A, Pérez-López C, Rodríguez-Martín D, Català A, Moreno-Aróstegui JM, Cabestany J, de Mingo E, Rodríguez-Molinero A (2017). Estimating bradykinesia severity in Parkinson's disease by analysing gait through a waist-worn sensor. Comput Biol Med.

[24] Rodriguez-Martin D, Samà A, Perez-Lopez C, Català A, Cabestany J, Rodriguez-Molinero A (2013). SVM-based posture identification with a single waist-located triaxial accelerometer. Expert Syst Appl.

[25] Horne MK, McGregor S, Bergquist F (2015). An objective fluctuation score for Parkinson's disease. PLoS One.

[26] Patel S, Lorincz K, Hughes R, Huggins N, Growdon J, Standaert D, Akay M, Dy J, Welsh M, Bonato P (2009). Monitoring motor fluctuations in patients with Parkinson's disease using wearable sensors. IEEE Trans Inf Technol Biomed.

[27] Cancela J, Pansera M, Arredondo MT, Estrada JJ, Pastorino M, Pastor-Sanz L, Villalar JL (2010). A comprehensive motor symptom monitoring and management system: the bradykinesia case. Conf Proc IEEE Eng Med Biol Soc.

[28] Rodríguez-Molinero A, Samà A, Pérez-López C, Rodríguez-Martín D, Alcaine S, Mestre B, Quispe P, Giuliani B, Vainstein G, Browne P, Sweeney D, Moreno AJ, Bayes À, Lewy H, Costa A, Annicchiarico R, Counihan T, Laighin GÒ, Cabestany J (2017). Analysis of correlation between an accelerometer-based algorithm for detecting Parkinsonian gait and UPDRS subscales. Front Neurol.

